# Molecular cloning and heterologous expression analysis of *JrVTE1* gene from walnut (*Juglans regia*)

**DOI:** 10.1007/s11032-015-0414-2

**Published:** 2015-11-17

**Authors:** Cancan Wang, Chuanrong Li, Charles A. Leslie, Qingrong Sun, Xianfeng Guo, Keqiang Yang

**Affiliations:** College of Forestry, Shandong Agricultural University, Taian, 271018 Shandong Province People’s Republic of China; Shandong Taishan Forest Ecosystem Research Station, Taian, 271018 People’s Republic of China; Department of Plant Sciences, University of California-Davis, Davis, CA 95616 USA; Shandong Institute of Pomology, Taian, 271018 Shandong Province People’s Republic of China

**Keywords:** *Juglans regia*, *JrVTE1*, Genetic transformation, *Zizyphus jujuba* var. *spinosa*, *Pyrus communis*, Tocopherols

## Abstract

Tocopherol cyclase (VTE1) plays a key role in promoting the production of γ-tocopherol and improving total tocopherol content in photosynthetic organisms. Walnut is an important source of tocopherols in the human diet, and γ-tocopherol is the major tocopherol compound in walnut kernels. In this study, a full-length cDNA of the *VTE1* gene was isolated from walnut using RT-PCR and RACE, and designated as *JrVTE1*. The full-length cDNA of the *JrVTE1* gene contained a 1353-bp open-reading frame encoding a 451-amino-acid protein with a calculated molecular weight of 49.5 kDa. The deduced JrVTE1 protein had a considerable homology with other plant VTE1s and belonged to the tocopherol cyclase family. Functional characterization of *JrVTE1* by heterologous expression was carried out in *E. coli* BL21 (DE3) and microshoot lines of the fruit trees jujube (*Zizyphus jujuba* var. *spinosa*) and pear (*Pyrus communis*) cultivar ‘Old Home’. *JrVTE1* in *E*. *coli* expressed as a 50 kDa protein, as expected. One or two copies of the transferred *JrVTE1* gene were detected in the genomes of representative transgenic lines (from the initial transgenic plants) of jujube and pear by gel blots analysis. Over-expression of *JrVTE1* in jujube and pear resulted in an accumulation of tocopherol and a shift in tocopherol composition in leaf, root and stem tissues. In the transgenic jujube, the total tocopherol content increased by 29.8 μg/g in the stems of line J3, 43.7 and 22.5 μg/g in the roots and leaves of line J1, respectively, whereas in the transgenic pear it increased by 47.3 μg/g in the leaf of line P3, and 16.7 and 10.4 μg/g in roots and stems of line P9, respectively. In the examined tissues of transgenic plants, the highest accumulation rate was the γ-tocopherol. These results indicate that *JrVTE1* is one of the rate-limiting enzymes for tocopherol production and could be used to improve the tocopherol content of tree crops through genetic engineering.

**Electronic supplementary material:**

The online version of this article (doi:10.1007/s11032-015-0414-2) contains supplementary material, which is available to authorized users.

## Introduction

Vitamin E, a group of minor but ubiquitous lipid-soluble compounds, consists of four forms of tocopherol (α-, β-, γ-, δ-tocopherol) and tocotrienol (α-, β-, γ-, δ-tocotrienol). Among these, α-tocopherol (αT) was considered the most biologically active form and had captured much attention (Jiang et al. 2004; Amaral et al. 2005). However, several studies have shown that γ-tocopherol (γT) may induce cell death in human prostate cancer cells and induce human breast cancer cells to undergo apoptosis (Jiang et al. 2004; Yu et al. 2008; Hassim et al. 2014). Lee et al. (2009) found that γ- and δ-tocopherols, but not α-tocopherol, activated peroxisome proliferator-activated receptor-γ and antagonized estrogen action in breast cancer.

Vitamin E, synthesized only by photosynthetic organisms, is obtained mainly from vegetable oils, nuts, cereals, green vegetables, fruits and oil seeds, and γ-tocopherol is the major form in seeds (Amaral et al. 2005; Gilliland et al. 2006). Previously, five enzymes (VTE1, VTE2, VTE3, VTE4 and VTE5) functioning in the tocopherol biosynthetic pathway have been elucidated in *Arabidopsis* (Addlesee et al. 1996; Collakova and DellaPenna 2001; Porfirova et al. 2002; Bergmüller and Dörmann 2003; Sattler et al. 2003; Valentin et al. 2006). Among them, the tocopherol cyclase (TC, VTE1) has been reported to be the key enzyme that catalyzes conversion of 2,3-dimethyl-6-phytyl-1,4-benzoquinone (DMPBQ) to γ-tocopherol and promotes the production of γ-tocopherol and the total vitamin E content (Porfirova et al. 2002; Cheng et al. 2003; Kanwischer et al. 2005; Vidi et al. 2006). Hence, much effort has been expended in overexpressing *VTE1* to increase γ-tocopherol production and vitamin E content in plants such as *Arabidopsis* (Kanwischer et al. 2005), transgenic rapeseed (Kumar et al. 2005), transgenic lettuce (Lee et al. 2007) and transgenic tobacco (Yabuta et al. 2013).

Walnut (*Juglans regia*), an excellent source of many nutrients (Isabel et al. 2013), has high oil, protein, vitamin and mineral content (Ma et al. 2010) and has been proposed as a promising natural food. The beneficial effects of walnut consumption, based on their vitamin E activity, have been well documented (Sze-Tao and Sathe 2000; Mène-Saffrané and DellaPenna 2010; Isabel et al. 2013). γ-Tocopherol has been found to be the major vitamin in walnuts (Lavedrine et al. 1997; Amaral et al. 2005), but only a few studies have been conducted on identifying the enzymes which limit the synthesis of γ-tocopherol (Kanwischer et al. 2005; Kumar et al. 2005; Lee et al. 2007; Yabuta et al. 2013). In this study, we aimed to isolate the *JrVTE1* gene from the developing embryo of walnut cultivar ‘*Xiangling*’ and to accomplish functional characterization of *JrVTE1* by heterologous expression analysis in *E. coli* BL21 (DE3), and in microshoot lines of the woody plants, jujube (*Zizyphus jujuba* var. *spinosa*) and pear (*Pyrus communis*) cultivar ‘Old Home’. We also expect to improve the tocopherols content of tree crops through genetic engineering.

## Materials and methods

### Plant materials

The *JrVTE1* gene was isolated from a developing walnut embryo at 90 days after flowering (DAF). The nut was taken from a 10-year-old tree of walnut cultivar ‘*Xiangling*’ grown at the forestry experimental station of Shandong Agricultural University, Taian, Shandong Province, China. Microshoot lines of jujube (*Z*. *jujuba* var. *spinosa*) and pear (*P*. *communis*) cultivar ‘Old Home’ were obtained from the Shandong Institute of Pomology, Taian, Shandong Province, China, and used for genetic transformation to characterize the function of *JrVTE1.*

### Isolation of *JrVTE1* gene from walnut

Total RNA was extracted from the developing walnut embryo following a modified CTAB method (Xu et al. 2012). The partial *VTE1* cDNA sequence was amplified by nested polymerase chain reaction (PCR) with a one-step RT-PCR kit (TaKaRa, Dalian, China) using degenerate oligonucleotide primers NGSPF and NGSPR (Table S1). The PCR product was purified using a TaKaRa MiniBEST Gel Extraction Kit (TaKaRa) and ligated into the pMD18-T vector (TaKaRa, Dalian, China) for sequencing at Sangon Biotech (Shanghai, China). Based on the partial *VTE1* cDNA sequence, the specific primers GSR3 and GSR5 (Table S1) were designed to perform 5′ rapid amplification of cDNA ends (RACE), and 3′ RACE reactions using the SMARTTM RACE cDNA Amplification kit (Takara, Clontech, China). The 5′ end and 3′ end cDNA sequences were assembled to obtain the full-length cDNA sequence by inserting into a pMD18-T vector for sequencing. Primers JrVTE1-FLF and JrVTE1-FLR (Table S1) were designed to generate the full-length cDNA of *JrVTE1* using 5′-RACE-Ready cDNA as a template. The PCR product was inserted into the pMD18-T vector for sequencing, and the vector was named as pMD18-T-*JrVTE1.*

The identity of walnut *JrVTE1* gene was confirmed via the nucleotide-nucleotide basic local alignment search tool (BLASTn) in the NCBI database. The sequence of *JrVTE1* gene was aligned with thirteen of known homologous genes from other plant species by ClustalW (Thompson et al. 1997; Larkin et al. 2007). The neighbor-joining tree (NJ) was constructed based on the p-distance in software MEGA5 (Tamura et al. 2007, 2011). DNAMAN version 4.0 software (Lynnon Biosoft, USA) was used for the deduced amino acid sequences analysis and assembly. pI/Mw Tool software at ExPaSy (http://web.expasy.org/compute_pi/) was used to predict the calculated molecular weight of the deduced JrVTE1.

### Quantification of *JrVTE1* transcripts in walnut embryos

The quantification of *JrVTE1* transcription in walnut embryos at 60, 90 and 120 DAF was determined by real-time quantitative PCR (RT-qPCR). Primers JrVTE1-YF and JrVTE1-YR, ACTINF and ACTINR (Table S1) were designed to amplify a common fragment shared by the cDNA sequence of *JrVTE1* and the sequence of the β-actin gene (a house-keeping gene). Total RNA was isolated from each walnut embryo and treated with RNase-free DNase I at 37 °C for 30 min using the DNase I kit (TaKaRa, Dalian, China). For RT-qPCR, samples were analyzed using a Bio-Rad iQ5 real-time PCR detection system with iQ5 optical system software (Carlson et al. 2008). Each reaction volume was 50 μL: 4 µL DNA (~400 ng), 2 µM of each primer, 25µL SYBR *Premix ExTaq*™II (TaKaRa, Dalian, China) and sterile water added to the final volume. The PCR conditions were at 95 °C for 3 min, 40 cycles at 94 °C for 20 s, 53 °C for 20 s and 72 °C for 2 min. Each sample was replicated three times and reactions with no template were used as negative control.

### Expression of *JrVTE1* in *E. coli*

The open-reading frame (ORF) of *JrVTE1* was amplified from pMD18-T-*JrVTE1* using the primers JrVTE1-OF and JrVTE1-OR (Table S1) with *Bam*HI and *Hin*dIII restriction sites at the 5′ and 3′ ends, respectively. The amplified DNAs were cloned into a pMD18-Simple vector (TaKaRa, Dalian, China) for sequencing, and this vector was named pMD18-Simple-*JrVTE1. Bam*HI and *Hin*dIII were used to digest both pMD18-Simple-*JrVTE1* and the prokaryotic expression vector pET-28a (preserved in our laboratory). Then, vector pET-28a-*JrVTE1* was created by cloning the digested product *JrVTE1* into corresponding region of the pET-28a expression vector and confirmed by restriction enzyme digestion and sequencing. Finally, pET-28a-*JrVTE1* was transformed into *E. coli* BL21 (DE3) competent cells (TransGen Biotech, Beijing, China), in which the expression of the JrVTE1 protein was induced by 1 mM isopropyl-beta-d thiogalactopyranoside (IPTG) at different induction times (0, 2, 4, 6, 8 and 10 h). The protein was analyzed on a 12.5 % SDS-PAGE with the empty vector serving as a control.

### Genetic transformation of *JrVTE1* in jujube and pear

Plant expression vector construction: The ORF of *JrVTE1* was amplified from pMD18-T-*JrVTE1* using the primers JrVTE1-ZOF and JrVTE1-ZOR (Table S1) with *N*de I and *S*al I restriction sites at the 5′ and 3′ ends, respectively. The amplified DNAs were cloned into a pMD18-Simple vector (TaKaRa, Dalian, China) and sequenced. The resulting vector was named pMD18-Simple-Z*JrVTE1. N*de I and *S*al I were used to digest both pMD18-Simple-Z*JrVTE1* and the plant expression vector pRI101 (TaKaRa, Dalian, China). Finally, *JrVTE1* was cloned into vector pRI101 to create vector pRI101-*JrVTE1* and confirmed by sequencing.

Genetic transformation: pRI101-*JrVTE1* was transformed into *Agrobacterium tumefaciens* (strain AGL1) and used to transform microshoot lines of jujube (*Z*. *jujuba* var. *spinosa*) and pear (*P*. *communis*) cultivar ‘Old Home’ by the leaf transformation method described by Sun et al. (2011). The calli of jujube and pear produced excisable shoots (2–3 mm) after a period of 7–8 months on selection medium. The marker gene neomycin phosphotransferase-II (*Npt*II) was used to select the transgenic plants. Later roots were induced from regenerated explants on rooting medium. The transgenic jujube and pear were grown in a culture room at 25 °C with a photoperiod of 16-h light and 8-h darkness. They were transferred to pots and acclimated for 2 weeks in the culture room and then moved to the greenhouse.

### Identification of transgenic lines

PCR analysis: Transgenic plants were confirmed by PCR using the primers JrVTE1-ZOF and JrVTE1-ZOR (Table S1). The PCR conditions were at 95 °C for 3 min, 30 cycles at 94 °C for 20 s, 54 °C for 20 s and 72 °C for 2 min, and the last step was at 72 °C for 5 min. The wild types were used as the control.

Southern blot analysis: Ten μg of genomic DNA from each transgenic plant was digested at 37 °C overnight using HindIII restriction enzyme (TaKaRa, Japan), subjected to electrophoresis on 0.7 % agarose gel and transferred to positively charged nylon membrane (Amersham, USA). Probe labeling, pre-hybridization, hybridization and subsequent luminescent detection were performed using the DIG High Prime DNA Labeling and Detection Starter Kit II following the protocol (Roche, USA). Twenty µL of denatured DIG-labeled probe was added to the blots and was incubated at 37 °C overnight. Blots were washed and the hybridization signals were detected by chemiluminescent detection following the manufacturer’s suggested protocol for DIG/CDP-Star (Roche, USA). Blots were exposed to X-ray films for 1–3 h at room temperature, and autoradiograms were developed (Dandekar et al. 1998; Kumar et al. 2015).

### Determination of tocopherols by HPLC

Standard and sample preparation: Tocopherol (α-, β-, γ- and δ) (Sigma-Aldrich Co., China) was used as internal standard (IS). A stock solution of the IS (10 mg/mL) was prepared in *n*-hexane, kept at −20 °C and protected from light, and the final concentration of standard α-, β-, γ- and δ-tocopherols was 1 mg/mL.

One gram of finely ground walnut was accurately weighed and introduced into glass test tubes with 100 μL of butylated hydroxytoluene (BHT) solution (10 mg/mL) as antioxidant (Amaral et al. 2005), and the extraction was extracted with 20 mL *n*-hexane by ultrasound for 15 min. Extracts were evaporated until dried in a vacuum rotoevaporator (Ya Rong, Shanghai) at 37 °C. The residue was dissolved in 20 mL *n*-hexane, filtered (0.22 μm membrane) and into 1.5 mL autosampler tubes.

One gram of each tissue (leaf, root and stem) from transgenic and non-transgenic jujube and pear was extracted with 10 mL *n*-hexane by ultrasound for 15 min. One gram of anhydrous sodium was added and then vortexed immediately and vigorously for 1.5 min. After centrifugation for 5 min at 5000 revolutions per minute (rpm), the supernatant (5 mL) was concentrated to 1 mL and filtered (0.22 μm membrane) into 1.5-mL autosampler tubes.

Determination of tocopherols: Tocopherols were separated by normal-phase HPLC (90 % [v/v] *n*-hexane and 10 % methyl tertbutyl ether) using an injection volume of 20 μL, a flow rate of 1.0 mL/min, and a run time of 20 min. HPLC analysis was performed on a LiChrospher Si-60 column (5 μm) (Merck, Germany) (Pinheiro-Sant’ana et al. 2011), using a UV detector (Shimadzu, 10AVP, Japan). The detection wavelength was set at 285 nm. Peaks of α-, β-, γ- and δ-tocopherol were identified by comparing their retention times with commercially available authentic standards. Tocopherol concentration and composition was calculated from standard curves.

## Results

### Tocopherol content in the developing walnut embryo

HPLC measurements showed that α-, β-, γ- and δ-tocopherols were all present in the developing walnut embryo (Supplement 2). Total tocopherol content was approximately 32, 96 and 108 µg/g in walnut embryos at 60, 90, and 120 DAF, respectively, and γ-tocopherol was the main form at each time period. The total tocopherol content at 90 DAF was twofold higher than at 60 DAF, and γ-tocopherol accumulated rapidly from 60 to 90 DAF but more slowly during the 90–120 DAF intervals (Supplement 2).

### Isolation and phylogenetic analysis of the *JrVTE1* gene

The full-length cDNA of the *JrVTE1* gene was isolated and designated as *JrVTE1* (GenBank accession no. KC751543) which contained a 1353 bp ORF encoding a 451-amino-acid protein with a calculated molecular weight of 49.5 kDa.

Alignment of the deduced amino acid sequences of *VTE1* homologs from *Glycine max* (XP_003522704) and other plant species showed that JrVTE1 shared 72 % identity with *Eucalyptus gunnii* and *Glycine max*, 71 % with *Hevea brasiliensis* and *Vitis vinifera*, 69 % with *Sesamum indicum*, and 67 % with *Brassica napus* (Supplement 3).

**Fig. 1:**
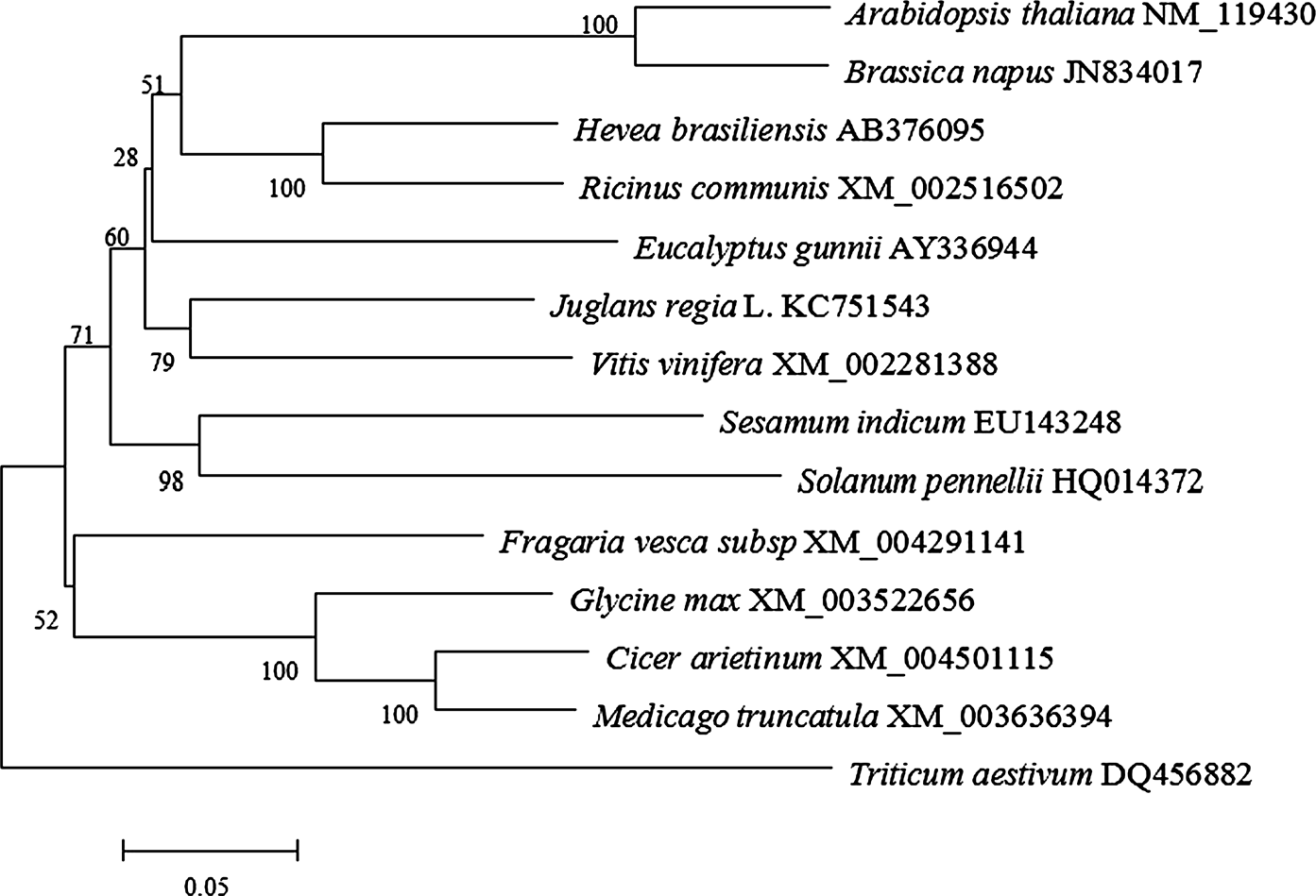
Phylogenetic analysis of the ORF sequences of *JrVTE1* and other plant species by MEGA 5. A phylogenetic tree of *VTE1* homologs constructed by the neighbor-joining method with p-distance

The neighbor-joining tree (NJ) showed that the *VTE1s* formed three clades with Clade 1 consisting of *VTE1s* from nine genera, including *Arabidopsis*, *Eucalyptus* and *Solanum*. Clade 2, a sister to clade 1, was comprised of *VTE1s* from species of the legume family such as *Glycine max*, *Medicago truncatula* and *Cicer arietinum*. *VTE1* from *Triticum aestivum*, a monocot, was more distantly related. *JrVTE1* was a sister to *Vitis vinifera*, being nested into Clade 1, indicating a common ancestor with other *VTE1s* (Fig. 1). These all indicated that JrVTE1 protein had a considerable homology with other plant VTE1s which belong to the tocopherol cyclase family.

### *JrVTE1* gene expression in the developing walnut embryo

To investigate *JrVTE1* gene expression at different periods during development of the walnut embryo, total RNA was isolated from embryos at 60, 90 and 120 DAF. Real-time PCR analysis revealed that *JrVTE1* transcripts were present in all tested samples with the greatest amount of transcript detected at 90 DAF (Supplement 6).

### *JrVTE1* expression in prokaryotic cells

When recombinant expression plasmid pET28a-*JrVTE1* was transferred into *E. coli* and expression of the target protein was induced by addition of IPTG to cells cultured for 0–10 h, a protein of the expected 49.5 kDa molecular weight was expressed and increased in concentration with increasing induction time. This protein was absent in non-induced cells transformed with the same vector. The target protein did not appear in cells transformed with the negative control plasmid pET-28a during any of the culture periods (Supplement 7). Expression of the target protein maximized at ~6 to 10 h (Supplement 7).

### Genetic transformation

**Fig. 2:**
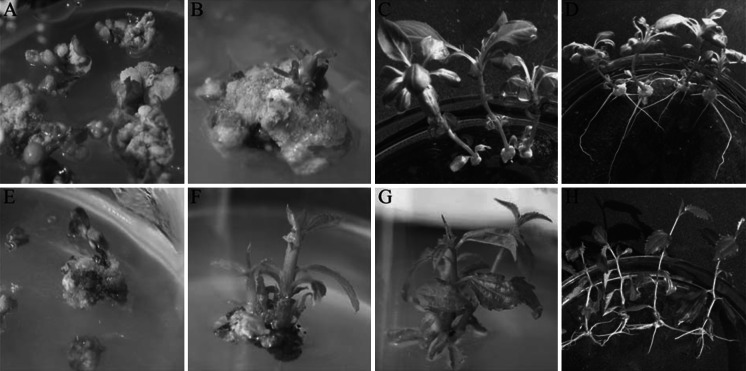
Plant regeneration from jujube and pear transformed with *JrVTE1*. **a**, **e** Putative transgenic adventitious calli from leaves of pear and jujube, respectively. **b**, **f** Putative transgenic adventitious bud regeneration from leaf calli of pear and jujube, respectively. **c**, **g** Transgenic shoot proliferation of pear and jujube, respectively. **d**, **h** Transgenic plants of pear and jujube, respectively

Approximately 35 and 65 % of the jujube and pear calli produced excisable shoots (2–3 mm) on selection medium. Fifteen transgenic lines of jujube and forty-one transgenic lines of pear were obtained. The plant regeneration from transformed pear and jujube lines are presented in Fig. 2.

### Confirmation of transgenic lines

PCR analysis: Genome DNA extracted from each putative transgenic line of jujube and pear was used as the template for PCR analysis. Transgenic lines J1, J3, J4, J6, J8 and J9 of jujube and transgenic lines P3, P7, P9, P10, P12, P15, P16 and P18 of pear were all detected the DNA fragment of 1353 bp; No amplification product was detected from DNA samples of transgenic lines P4, P6 and the wild types JW, PW (Supplement 8).

**Fig. 3:**
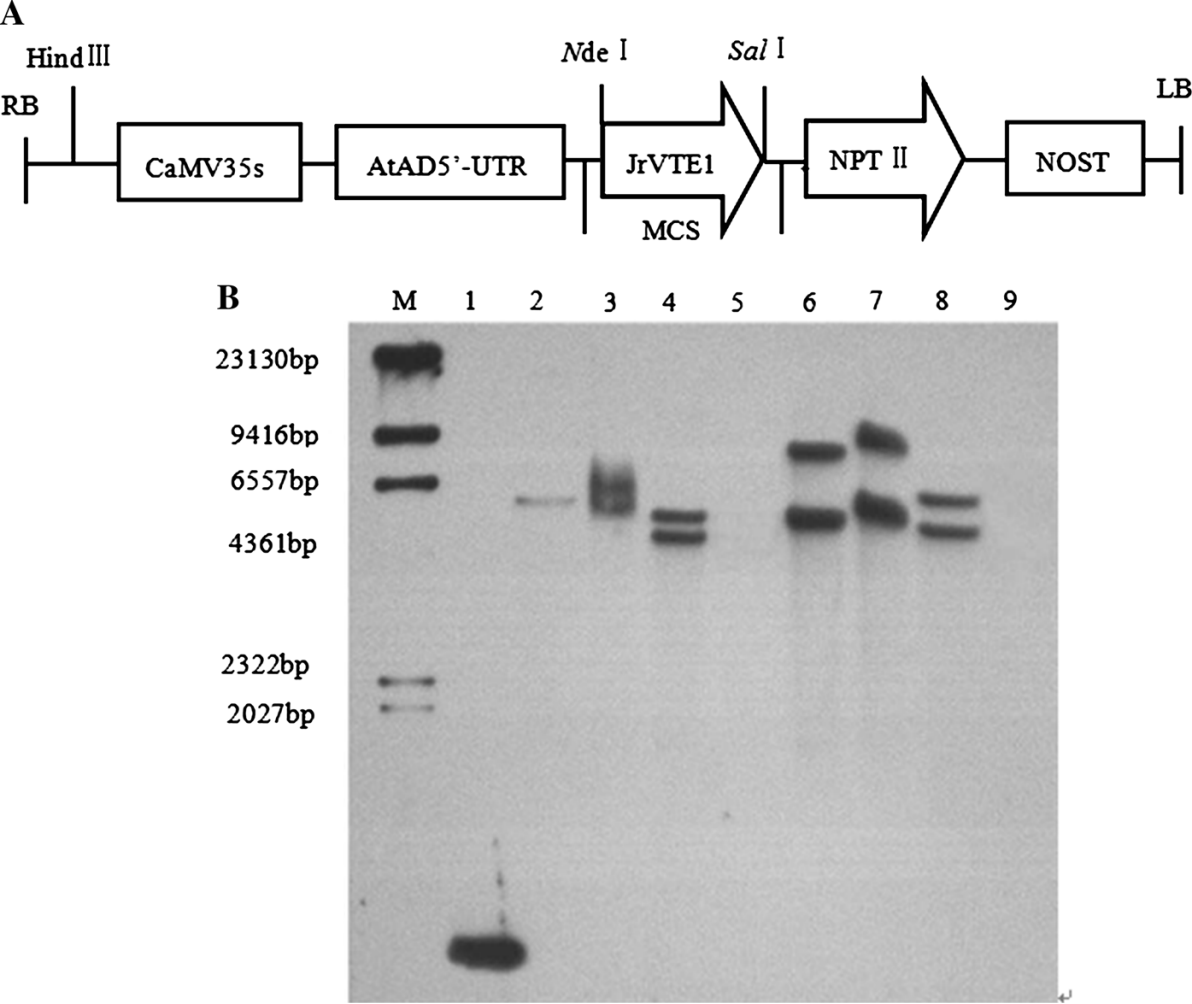
Southern blot analysis of six representative transgenic lines. **a** Schematic representation of T-DNA region of binary vectors pRI101. RB: right border, CaMV35S: cauliflower mosaic virus 35S promoter, AtAD5′-UTR: the enhancer of *JrVTE1* expression, *JrVTE1*: tocopherol cyclase gene, NOST: nopaline synthase gene terminator, NPTII: neomycin phosphotransferase II, LB: left border. **b** Southern blot analysis of transgenic jujube and pear lines. *lane 1* is the positive control: PCR product of *JrVTE1* (1.3 kb) gene, *lanes 2*, *3*, and *4* show the presence of *JrVTE1* integration in transgenic jujube lines 4, 3, and 1, *lane 5* is wild jujube DNA not showing any band, *lanes 6*, *7*, and *8* show the presence of *JrVTE1* integration in transgenic pear lines 3, 7 and 9, *lane 9* is wild pear DNA not showing any band

Southern hybridization analysis: Integration of the transgene-bearing T-DNA (Fig. 3a) into the recipient jujube and pear genomes was further confirmed by Southern hybridization analysis. Gel blots of *Hin*dIII-digested genomic DNA of three representative transformants of jujube and pear gave different hybridization bands, and no band was found in the wild type (Fig. 3b). The transgenic lines J1, J3, J4 of jujube and lines P3, P7, P9 of pear all arose from independent transformation events, and the genomic loci into which the T-DNA was inserted were likely different. Two copies of the transferred *JrVTE1* gene were detected in the genomes of lines J1, J3, P3, P7, P9 and one copy in line J4 of jujube.

### Functional analysis of *JrVTE1*

**Fig. 4:**
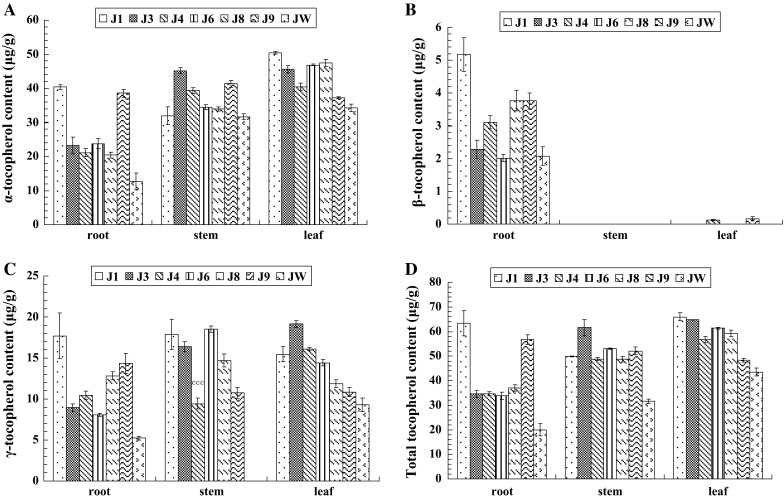
Analysis of tocopherol content in transgenic jujube roots, stems and leaves. **a** The α-tocopherol content (μg/g) in roots, stems and leaves of wild-type (JW) and transgenic 35S::*JrVTE1* jujube lines (J1, J3, J4, J6, J8 and J9). **b** The β-tocopherol contents (μg/g) in roots, stems and leaves of wild-type (JW) and transgenic 35S::*JrVTE1* jujube lines (J1, J3, J4, J6, J8 and J9). **c** The γ-tocopherol content (μg/g) in roots, stems and leaves of wild-type (JW) and transgenic 35S::*JrVTE1* jujube lines (J1, J3, J4, J6, J8 and J9). **d** The total tocopherol content (μg/g) in roots, stems and leaves of wild-type (JW) and transgenic 35S::*JrVTE1* jujube lines (J1, J3, J4, J6, J8 and J9). The experiments were independently replicated three times

**Fig. 5:**
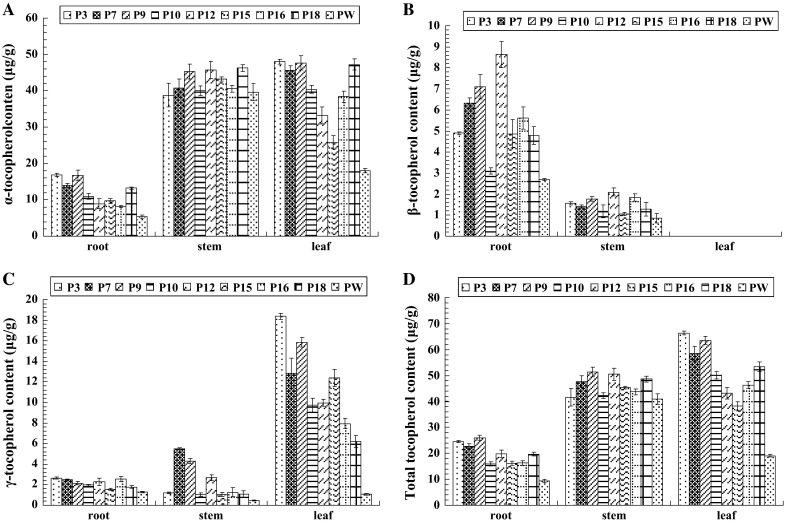
Analysis of tocopherol content in transgenic pear roots, stems and leaves. **a** The α-tocopherol content (μg/g) in roots, stems and leaves of wild-type (PW) and transgenic 35S::*JrVTE1* pear lines (P3, P7, P9, P10, P12, P15, P16 and P18). **b** The β-tocopherol contents (μg/g) in roots, stems and leaves of wild-type (PW) and transgenic 35S::*JrVTE1* pear lines (P3, P7, P9, P10, P12, P15, P16 and P18). **c** The γ-tocopherol contents (μg/g) in roots, stems and leaves of wild-type (PW) and transgenic 35S::*JrVTE1* pear lines (P3, P7, P9, P10, P12, P15, P16 and P18). **d** The total tocopherol contents (μg/g) in roots, stems and leaves of wild-type (PW) and transgenic 35S::*JrVTE1* pear lines (P3, P7, P9, P10, P12, P15, P16 and P18). The experiments were independently replicated three times

HPLC analysis showed that overexpression of *JrVTE1* results in accumulation of tocopherol and a shift in tocopherol composition in each tissue of transgenic jujube and pear examined. Total tocopherol and α-tocopherol were found mainly in the leaves of both of jujube and pear, not in roots and stems, and δ-tocopherol was not detected in any tissue (Figs. 4, 5). Overexpression of *JrVTE1* using a 35S promoter resulted in an increase of 29.8 μg/g in total tocopherol in the stems of the jujube transformant line J3, and an increase of 43.7 and 22.5 μg/g in the roots and the leaves of line J1, respectively (Fig. 4d). The γ-tocopherol content in the roots of the transformants was 53 % ~2.3-fold higher than wild type (JW) (Fig. 4c). In the pear transformant line P9, total tocopherol content was increased by 16.7 and 10.4 μg/g in roots and stems, respectively, and 47.3 μg/g increased in the leaf of line P3 (Fig. 5d). The γ-tocopherol content in the leaves of the transformants was almost 4.8–16.2-fold higher than wild type (PW) (Fig. 5c).

## Discussion

### *JrVTE1* gene was isolated from walnut and belonged to the tocopherol cyclase family

The aim of forest tree genomics is to support genetic improvement programs via understanding the association between gene function and morphological traits (Neale and Kremer 2011). The draft genome sequence of the cultivar ‘Chandler’ has already been produced (http://www.ncbi.nlm.nih.gov/bioproject/285351). And many functional genes have been isolated from walnut, such as a gene encoding a 2S albumin seed storage protein precursor (Teuber et al. 1998), two full orthologous cDNAs of chalcone synthase (CHS) which is the key enzyme of flavonoid biosynthesis (Claudot et al. 1999), the orthologous cDNAs of the *jrAG* and *jrAP3* genes which have shown a preferential expression in flowers (Breton et al. 2004), the walnut SDH (shikimate dehydrogenase) gene that is essential for gallic acid (GA) synthesis (Muir et al. 2011), the *JrPAL* (Phenylalanine ammonia lyase) gene which is the first key enzyme gene of the phenylpropanoid pathway (Xu et al. 2012), and Araji et al. (2014) suggested that the *jrPPO1* gene (enzyme polyphenol oxidase) plays a novel and a fundamental role in secondary metabolism and acts as an indirect regulator of cell death in walnut. However, walnut is a healthy dietary component with high micronutrient content and there is significant potential benefit in isolating the relevant genes in order to understand the molecular and biochemical basis of their impact on the micronutrient level of walnut (Gilliland et al. 2006). Hence, the full-length cDNA of the *VTE1* gene, which plays a key role in promoting the production of γ-tocopherol and improving total tocopherol content in photosynthetic organisms, was successfully isolated from the developing embryo of the walnut cultivar ‘*Xiangling*’. Sequence multi-alignment demonstrated that the deduced JrVTE1 protein had a considerable homology to other plant VTE1s and belonged to the tocopherol cyclase family (Supplement 3). Phylogenetic analysis showed that *JrVTE1* has a common ancestor with other *VTE1s* and is a sister to the *VTE1* from *Vitis vinifera* (Fig. 1).

### The dynamics of γ-tocopherol accumulation are consistent with *JrVTE1* expression in walnut developing embryo

The ‘*Xiangling*’ walnut cultivar is popular in China. During its embryonic developmental process, fat and soluble sugar content peaks and protein metabolism is most active at 60–100 DAF. Fat content increases from 5.1 % at 60 DAF to 50.11 % at 100 DAF (Li et al. 2012). The total tocopherol content of the developing ‘*Xiangling*’ embryo at 60, 90 and 120 DAF is shown in Supplement 2, and γ-tocopherol is the main type of tocopherol present. γ-Tocopherol accumulated rapidly from 60 to 90 DAF and then slowly during the remaining 90–120 DAF. Similar trends have been observed in developing rapeseed seeds: γ-Tocopherol accumulated slowly over 12–41 DAF, and the greatest γ-tocopherol accumulation occurred from 41 to 53 DAF. For the rapeseed, both total tocopherol content and α- to γ-tocopherol ratios remained constant from 53 DAF to seed maturity (Goffman et al. 1999). Real-time PCR showed that *JrVTE1* gene transcripts could be detected in all walnut samples at 60, 90 and 120 DAF, with the highest transcript occurrence at 90 DAF (Supplement 6). Similarities between γ-tocopherol accumulation and *JrVTE1* expression in the walnut developing embryo suggest that γ-tocopherol accumulation is correlated with *JrVTE1* expression.

### *JrVTE1* expressed a 49.5 kDa expected protein in *E. coli*

To validate the reliability and accuracy of the gene cloned from walnut, plasmid pET-28a-*JrVTE1* was created and expressed in *E. coli* BL21 (DE3) competent cells. After the gene expression in *E. coli*., a protein of 50 kDa was detected as theoretically expected (49.5 kDa) (Supplement 7). This result was similar to that of the VTE1 from *Arabidopsis* in which the cyclase activity eluted with a molecular mass of about 50 kDa, corresponding to the calculated 47 kDa molecular mass (Kumar et al. 2005; Kanwischer et al. 2005). SDS-PAGE analysis showed that the content of the target protein increased as the induction time prolonged while the target protein was absent in non-induced cells (Supplement 7). The successful heterologous expression of *JrVTE1* gene in *E. coli* could lay a basis for further study of JrVTE1 protein function.

### Overexpression of *JrVTE1* results in an accumulation of tocopherol in jujube and pear mainly due to the increase in γ-tocopherol content

VTE1 catalyzes the cyclization of DMPQ resulting in the formation of γ-tocopherol that is subsequently converted to α-tocopherol (Cheng et al. 2003; Kanwischer et al. 2005; Vidi et al. 2006). Jujube and pear are important fruit crops with high commercial values (Nakajima et al. 2013; Liu et al. 2014). In this study, we successfully obtained the transgenic lines of jujube and pear using leaf as explants and employed the protocol of Sun et al. (2011), and detected one or two copies of the transferred *JrVTE1* gene in the representative transgenic lines of jujube and pear by gel blot (Fig. 3). We also found that in the transgenic jujube the total tocopherol content increased by 29.8 μg/g in the stems of the transgenic jujube line J3, 43.7 and 22.5 μg/g in the roots and leaves of line J1, respectively (Fig. 4), whereas in the transgenic pear the total tocopherol content increased by 47.3 μg/g in the leaf of line P3, and 16.7 and 10.4 μg/g in roots and stems of line P9, respectively (Fig. 5). And in the examined tissues of transgenic plants, the highest accumulation rate was the γ-tocopherol relative to wild type (Figs. 4, 5). Total tocopherol levels in leaf, root and stem of transgenic plants (jujube and pear) increased significantly due to an increase in γ-tocopherol, indicating that JrVTE1 is one of the rate-limiting enzymes for tocopherol production and γ-tocopherol accumulation in these tissues of transgenic plants. The commercial values of pear and jujube are in the fruits, so tocopherols content in the fruits of transgenic pear and jujube would be determined in our further work.

Previous studies reported that overexpression of the *Arabidopsis VTE1* gene in seeds of transgenic rapeseed plants, and in leaves of *Arabidopsis*, transgenic tobacco and transgenic lettuce, all resulted in an increase in total tocopherol, due mainly to an increase in γ-tocopherol (Kumar et al. 2005; Kanwischer et al. 2005; Lee et al. 2007; Yabuta et al. 2013; Harish et al. 2013). In addition, overexpressing *VTE1* can enhance tolerance to the environmental stresses. Liu et al. (2008) reported that overexpressing *VTE1* from *Arabidopsis* in tobacco could enhance tolerance to drought. Transgenic plants overexpressing *OsVTE1* from *Oryza sativa* showed higher tolerance to salt stress and increased the antioxidant capacity of rice seedlings (Ouyang et al. 2011). Therefore, abiotic stress tests of transgenic jujube and pear plants overexpressing *JrVTE1* could be used to identify superior lines which have wide adaptability to the environment as well as high content of tocopherols.

## Electronic supplementary material

Supplementary material 1 (DOC 31 kb)

Supplementary material 2 (XLS 32 kb)

Supplementary material 3 (TIFF 1124 kb)

Supplementary material 4 (TXT 7 kb)

Supplementary material 5 (TXT 25 kb)

Supplementary material 6 (XLS 25 kb)

Supplementary material 7 (DOC 3472 kb)

Supplementary material 8 (DOC 626 kb)

Supplementary material 9 (XLS 94 kb)

Supplementary material 10 (XLS 114 kb)
