# Supplementary material for: Molecular cloning and heterologous expression analysis of JrVTE1 gene from walnut (Juglans regia)
Source: Mol Breed. 2015 Nov 17;35:222. doi: 10.1007/s11032-015-0414-2 (PMC4648991; doi:10.1007/s11032-015-0414-2)
Supplement: Supplementary file 1 — Supplementary material 1 (DOC 31 kb) [file 11032_2015_414_MOESM1_ESM.doc]

**Table S1. Primers used in molecular cloning and expressing of *VTE1* gene.**

| **Primer Name** | **Primer Sequence（bp）** |
| --- | --- |
| NGSPF | 5'-TTYTTYGARGGITGGTA-3' |
| NGSPR | 5'-ATYTGCCARTGIGGYTC-3' |
| GSR 3 | 5'-CGAAGCCTCCATACAAGGAGGTCCC-3' |
| GSP 5 | 5'-AGGGCGGGTACTGTACTCCCAACGA-3' |
| JrVTE1-FLF | 5'-AGTCTTACTCGCTGATAGTCTTC-3' |
| JrVTE1-FLR | 5'-GTCAAATCTGTCGGCTAAAT-3' |
| JrVTE1-YF | 5'-TGGGCGCAAATGACAAG-3' |
| JrVTE1-YR | 5'-GACCTGCCATCATCACGAAT-3' |
| ACTINF | 5'-GCCGAACGGGAAATTGTC-3' |
| ACTINR | 5'-AGAGATGGCTGGAAGAGG-3' |
| JrVTE1-OF | 5'-CGCGGATCCATGTCGAGGGGC-3' |
| JrVTE1-OR | 5′-CGCAAGCTTCTAAAGACCAGGGGGT-3′ |
| JrVTE1-ZOF | 5'-CGCCATATG TCGAGGGGCCTATC-3' |
| JrVTE1-ZOR | 5'-CGCGTCGACCTAAAGACCAGGGGGT-3' |
