# Supplementary figures and images for: Molecular cloning and heterologous expression analysis of JrVTE1 gene from walnut (Juglans regia)
Source: Mol Breed. 2015 Nov 17;35:222. doi: 10.1007/s11032-015-0414-2 (PMC4648991; doi:10.1007/s11032-015-0414-2)

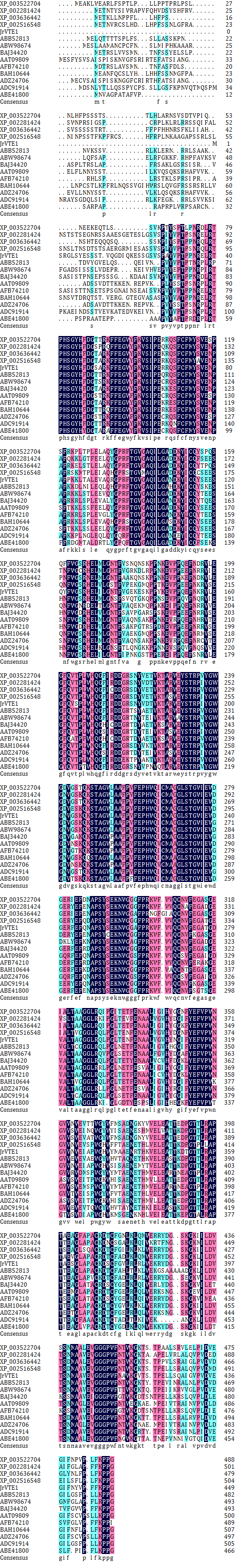

Supplement: Supplementary file 3 — Supplementary material 3 (TIFF 1124 kb) [file 11032_2015_414_MOESM3_ESM.tif]
