# Supplementary material for: Molecular cloning and heterologous expression analysis of JrVTE1 gene from walnut (Juglans regia)
Source: Mol Breed. 2015 Nov 17;35:222. doi: 10.1007/s11032-015-0414-2 (PMC4648991; doi:10.1007/s11032-015-0414-2)
Supplement: Supplementary file 7 — Supplementary material 7 (DOC 3472 kb) [file 11032_2015_414_MOESM7_ESM.doc]

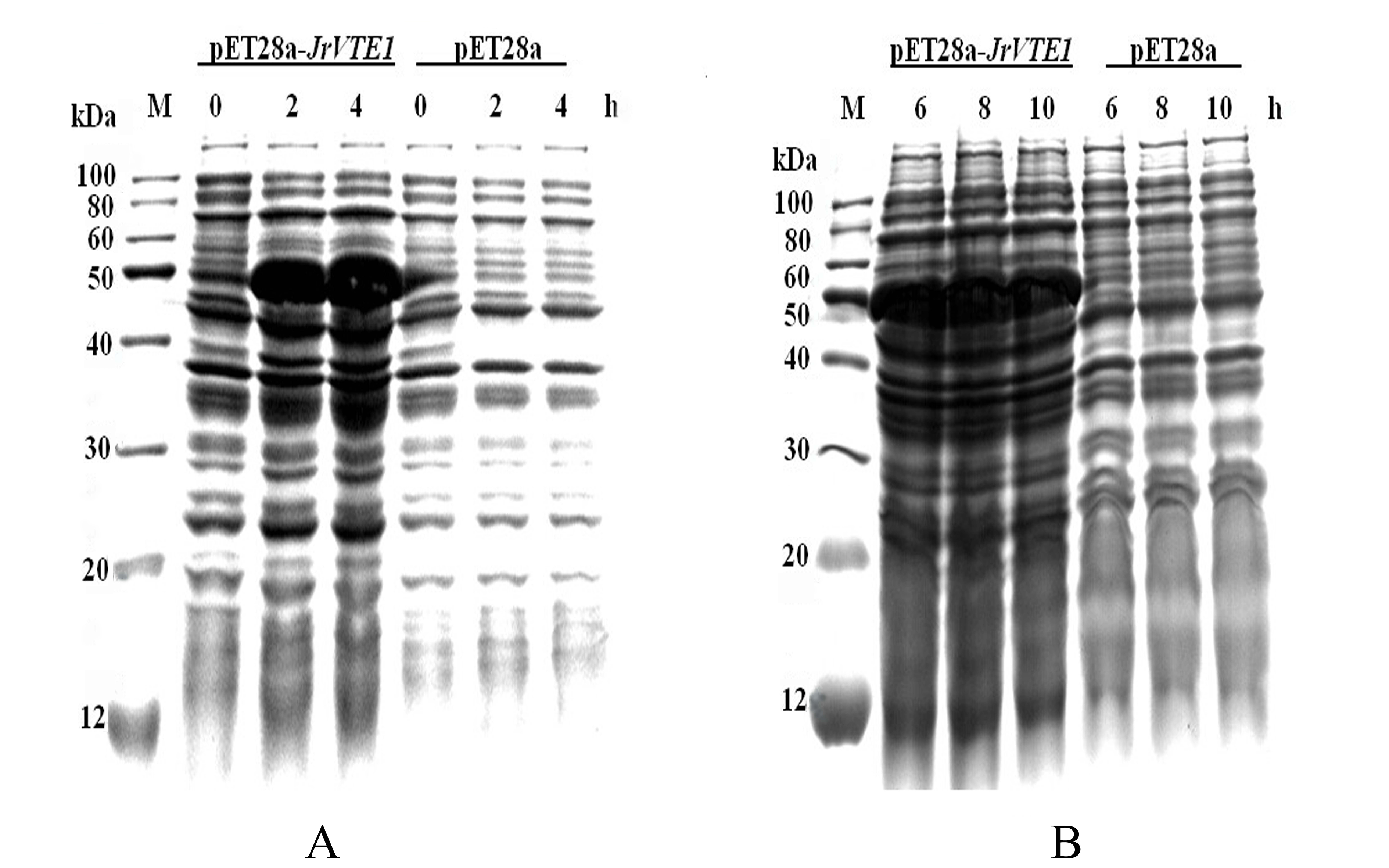


Figure S2. Expression of JrVTE1 in prokaryotic cells.

(A) Bacterial lysates of E. coli BL21 (DE3) cells containing pET-28a or recombinant plasmids were cultured for 0, 2 and 4 h respectively. (B) Bacterial lysates of E. coli BL21 (DE3) cells containing pET-28a or recombinant plasmids were cultured for 6, 8 and 10 h, respectively.
