# Supplementary material for: Molecular cloning and heterologous expression analysis of JrVTE1 gene from walnut (Juglans regia)
Source: Mol Breed. 2015 Nov 17;35:222. doi: 10.1007/s11032-015-0414-2 (PMC4648991; doi:10.1007/s11032-015-0414-2)
Supplement: Supplementary file 8 — Supplementary material 8 (DOC 626 kb) [file 11032_2015_414_MOESM8_ESM.doc]

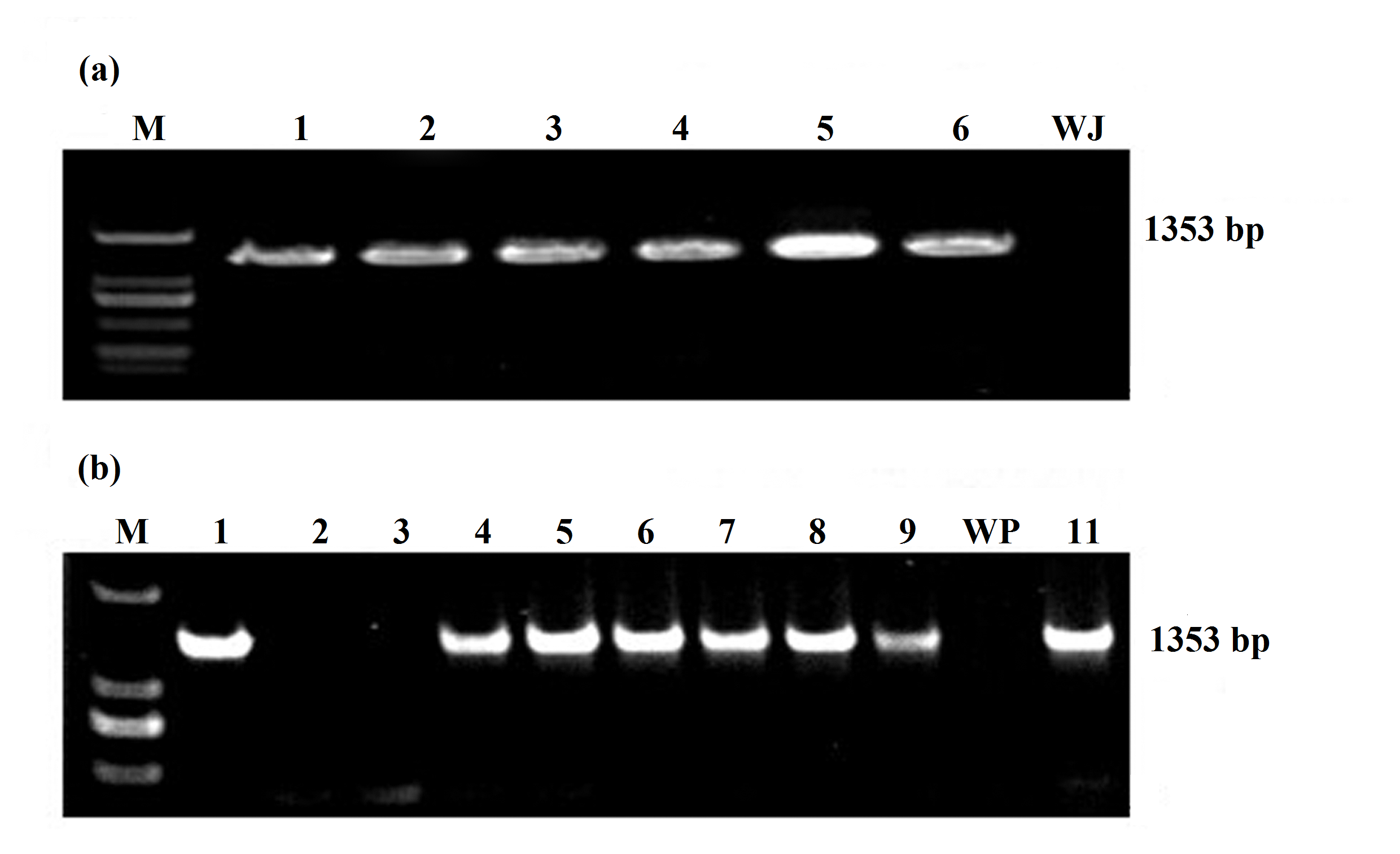


**Figure S3. PCR detection of wild-type and transgenic 35S::*JrVTE1* lines of jujube and pear.**

(A) PCR analysis of the wild-type and 35S::*JrVTE1* transgenic lines of jujube. Lane M is a 2kb DNA marker, Lanes 1 to 6 correspond to TC transgenic lines J1, J3, J4, J6, J8, J9, Lane JW is the wild type. (B) PCR analysis of wild-type and 35S:: *JrVTE1* transgenic lines of pear. Lane M is a 2kb DNA marker, Lanes 1 to 9, 11 correspond to TC transgenic lines P3, P4, P6, P7, P9, P10, P12, P15, P16, P18, Lane PW is the wild type.
